# Implications of gender-affirming endocrine care for sports participation

**DOI:** 10.1177/20420188231178373

**Published:** 2023-06-08

**Authors:** Ethan Moreland, Ada S. Cheung, Danielle Hiam, Brendan J. Nolan, Shanie Landen, Macsue Jacques, Nir Eynon, Patrice Jones

**Affiliations:** Institute for Health and Sport (IHeS), Victoria University, Footscray, VIC, Australia; Trans Health Research Group, Department of Medicine (Austin Health), The University of Melbourne, Heidelberg, VIC, Australia; Department of Endocrinology, Austin Health, Heidelberg, VIC, Australia; Institute for Physical Activity and Nutrition (IPAN), School of Exercise and Nutrition Sciences, Deakin University, Burwood, VIC, Australia; Trans Health Research Group, Department of Medicine (Austin Health), The University of Melbourne, Heidelberg, VIC, Australia; Department of Endocrinology, Austin Health, Heidelberg, VIC, Australia; Institute for Health and Sport (IHeS), Victoria University, Footscray, VIC, Australia; Institute for Health and Sport (IHeS), Victoria University, Footscray, VIC, Australia; Institute for Health and Sport (IHeS), Victoria University, Footscray, VIC, Australia; Institute for Health and Sport (IHeS), Victoria University, Ballarat Road, Footscray, VIC 3011, Australia

**Keywords:** gender-affirming hormone therapy, muscle, sports, transgender

## Abstract

Many transgender (trans) individuals utilize gender-affirming hormone therapy (GAHT) to promote changes in secondary sex characteristics to affirm their gender. Participation rates of trans people in sport are exceedingly low, yet given high rates of depression and increased cardiovascular risk, the potential benefits of sports participation are great. In this review, we provide an overview of the evidence surrounding the effects of GAHT on multiple performance-related phenotypes, as well as current limitations. Whilst data is clear that there are differences between males and females, there is a lack of quality evidence assessing the impact of GAHT on athletic performance. Twelve months of GAHT leads to testosterone concentrations that align with reference ranges of the affirmed gender. Feminizing GAHT in trans women increases fat mass and decreases lean mass, with opposite effects observed in trans men with masculinizing GAHT. In trans men, an increase in muscle strength and athletic performance is observed. In trans women, muscle strength is shown to decrease or not change following 12 months of GAHT. Haemoglobin, a measure of oxygen transport, changes to that of the affirmed gender within 6 months of GAHT, with very limited data to suggest possible reductions in maximal oxygen uptake as a result of feminizing GAHT. Current limitations of this field include a lack of long-term studies, adequate group comparisons and adjustment for confounding factors (e.g. height and lean body mass), and small sample sizes. There also remains limited data on endurance, cardiac or respiratory function, with further longitudinal studies on GAHT needed to address current limitations and provide more robust data to inform inclusive and fair sporting programmes, policies and guidelines.

## Introduction

Transgender (trans) is an umbrella term for individuals with a gender identity different from that presumed at birth and includes people with binary (man/woman) and non-binary identities. Gender affirmation, including social, legal and/or medical affirmation, plays an important role in improving the mental health of trans people.^1–3^ Medical affirmation involving gender-affirming hormone therapy (GAHT) results in clear changes in circulating sex hormone concentrations, which allows for the development of secondary sex characteristics more aligned to one’s affirmed gender.^4,5^ Feminizing GAHT typically involves oestradiol and anti-androgen administration, which induces breast formation, reduces male pattern hair growth, and alters fat and muscle distribution.^6,7^ Masculinizing GAHT involves testosterone therapy and stimulates increases in facial and body hair, changes in fat and lean mass distribution and longer-term effects such as deepening of the voice.^6,7^ While the effects of GAHT on secondary sex characteristics are well-established, the broader physiological effects of these hormone therapies in various tissues are less clear. In this respect, there is current interest in the impacts of GAHT on skeletal muscle and on measures relevant to participation, performance and safety in sports.

There is ongoing debate around whether trans individuals should be included in grass-roots and elite sport competitions. The premise of competitive sport, whether at the grass-roots and elite level, is based on subjective fairness, with the goal of inclusion for everyone, in the absence of discrimination.^8^ Many sports are separated into male and female categories due to evident sex differences in performance, and concerns for safety of female athletes in contact sports. On average, males are stronger than females and possess larger body sizes, muscle mass, and whole-body oxygen consumption.^9,10^ These differences contribute to differences in male and female performance, that range from 5 to 17%, depending on the sport and competition level.^11^ Due to limited evidence and research in trans health, it remains unclear whether there is an athletic advantage or disadvantage for trans men or trans women after undergoing GAHT in different sport disciplines. Participation in sport is a basic right for all people from any background. It is therefore important to understand whether GAHT confers any advantage or disadvantage in certain sport settings, to inform sports policies and programmes.^12^ Here, we review the current knowledge and limitations around the effects of GAHT on measures relevant to sports.

## Terminology

In this review, *male* and *female* are sex terms referring to someone’s sex presumed at birth. *Woman/women, man/men, non-binary* and *gender-diverse* are gender terms which describe a person’s gender identity. Someone who has a gender identity that aligns with their sex at birth may be referred to as *cisgender* (cis). *Transgender* (trans) describes people who have a gender identity different from the sex they were assigned at birth. This review focuses on studies of transgender men and women using standard doses of GAHT to achieve sex steroid concentrations in the range of the affirmed gender and does not cover people who may be using low doses of GAHT, given the lack of relevant studies.

## Brief view of transgender participation in sport

Trans athletes throughout history have undergone discrimination and exclusion in their given sports, despite trans people representing a small percentage of the general population (0.1–2.0%^13^) and sports competitors. As examples, trans women Renée Richards and Fallon Fox were banned from competing in their respective sports (1976: US Open Tennis Woman’s Championship, 2013: mixed martial arts) due to perceived performance advantages.^14,15^ The International Olympic Committee have allowed trans athletes to compete since 2003, provided certain criteria are met. In that time, despite over 70,000 individuals competing in the Olympic Games, only a handful of trans athletes have competed, and none have medalled; however, these athletes’ faced discrimination and public debate despite abiding by regulations and rules. More recently, in 2021, weightlifter Laurel Hubbard became the first openly trans woman to compete in an Olympics Game and was subjected to online abuse and media hounding.^16^ Trans men athletes have typically been the subject of less public debate; however, Chris Mosier, known for being the first trans man to compete in a men’s Olympic Trials in 2020, has spoken openly about the challenges faced to be eligible for these competitions.^17,18^

The increased visibility of trans people, in general, has fuelled ongoing public debates and policy actions around the inclusion of trans people in sport. Previous sporting guidelines have been largely focused on the serum testosterone concentrations of competing trans women athletes. In 2015, the International Olympic Committee (IOC) restricted many trans women athletes from competing in the women’s category by introducing a testosterone threshold of below 10 nmol/L for a minimum of 12 months prior to competition.^19^ Trans men were permitted to compete without restriction following 1 year of GAHT. However, in November 2021, the IOC released a new framework for participation, which encouraged moving away from testosterone-based restrictions and a ‘one-size-fits-all’ approach.^8^ The framework stated that sporting bodies should not assume that trans women have an inherent advantage over cis women, nor should they have to reduce their testosterone levels to compete. The IOC also concluded that individual sporting bodies should hold the responsibility of establishing guidelines for trans inclusion for their given sport, with guidelines based on robust and peer-reviewed evidence.^8^ While this approach makes logical sense, the issue is that it is practically impossible given the current lack of evidence in this field.

During the writing of this narrative review, FINA, the governing body of international competitive swimming, announced a 2022 policy stating that trans athletes who transitioned after puberty are ineligible to compete in elite international swimming competitions.^20^ FINA stated that the decision was made after consulting scientists and policymakers, yet there is currently no definitive research in trans swimmers which implies any performance enhancement in trans women. Sporting organizations currently appear pressured to make evidence-based policy decisions around trans participation, with the prevailing problem being there is very little quality research. Given that sport is an important fabric of society in many countries, there are also current concerns about how decisions being made at the elite sporting level will impact community sport guidelines and broader motivations of trans people to engage in sports.

At the community level, many sporting guidelines aimed at boosting the participation of trans people in sport have been recently introduced. For example, in 2020 in Australia, several governing bodies, including that of Tennis, Rugby, Australian Rules Football, Hockey, Netball, Water Polo, Touch Football and university sports released guidelines for trans participation.^21^ However, despite the recent development of trans-specific guidelines, participation in sports is exceedingly low.^22^ Recent studies reveal trans individuals engage less in sports and physical activity, when compared to cis individuals,^23^ and are deterred from competing in single-sex sports.^24,25^ A 2020 survey of Spanish trans individuals (*n* = 212) identified gender disclosure as a key issue in sports participation, with 14.5% stopping physical activity after gender disclosure,^24^ while it was reported 52% of boys and 44% of girls participated in sport,^26^ compared to 12% of trans youth (14% for trans boys and 12% for trans girls) participate in sports teams.^22^ This is a concern when considering that trans people are at higher risk of poor physical and mental health,^27,28^ and would especially benefit from regular engagement in sports and exercise. Given many organizational barriers to sports participation stem from the unclear effects of GAHT used by trans individuals, further research and critical review in this field is needed to inform updated policies, guidelines and sporting programmes which are evidence-based, fair and more inclusive and beneficial for all.

## Performance differences between sexes

Sex differences in performance between males and females are well reported and should be highlighted before discussions of differences in performance between cisgender individuals and trans individuals undergoing GAHT. A systematic review by Cheuvront *et al.* concluded that males held a considerable aerobic performance advantage over females, ranging from 8.4% (marathon distance) to 14.1% (5000 m).^29^ Performance advantages of 7.3–11.9% were also reported for males in sprint-based events of 100–1500 m.^29^ A 2007 review and comparison of the top male and female athletes in Olympic and World Championship events from 1952 to 2006 concluded that performance differences between sexes have widened since the 1990s in running, swimming and speed skating events.^30^ A study looking at swimming, running and jumping events reported the sex differences from the onset of male puberty to early adulthood. For track and field athletics, the study reported pre-pubertal sex differences from 3.0% to 10.1% across events. For jump performance, the difference between sexes pre-pubertal was ~5.8%, which increased to a 19.4% advantage for males after the onset of puberty.^31^ Sex differences are also seen in marathon events, with a 2018 study finding that females achieved their best marathon race times 5 years earlier than males, but males outperformed females in their best race times.^32^ An earlier study comparing marathon performance in matched males and females found significant differences in running economy, with females having poorer running economy and training volume over males.^33^ A 1998 review of the world’s best times in the 1500 m and marathon events from 1980 to 1996 states that sex difference in these events has plateaued at 11.2%.^34^

Performance differences between sexes are underpinned by sex variation in several measures, such as body composition, strength and aerobic capacity. In terms of strength, there is a large disparity in strength between adult males and females, which is a major reason for single-sex sporting competitions. Males are evidently stronger than females, with a comparative study concluding that females have 37–68% of the muscle strength of that of males, with the biggest disparity being in the upper body.^35^ As such, it would not be meaningful for males and females to compete together, particularly in impact sports. There are further sex differences in aerobic capacity, measured by maximal oxygen uptake (VO_2_max), with females having been found to have 70–75% of the capacity of males.^36^ This seems to be a significant factor in the differences in race times outlined above, particularly for middle and long-distance events.^37^ In terms of strength, it is well known that males have a greater capacity for maximal strength tasks such as weightlifting.^38^ One study performed two correlational observations, during which they found that in the first observation of national-level USA weightlifters, males (*n* = 39) displayed greater strength relative to bodyweight in the squat (22%), snatch (27%) and clean (28%) movements compared to females (*n* = 26).^38^ Males and females also differ in body composition,^39^ with one prospective study showing that males (*n* = 94) possessed 20% greater lean mass/weight ratio than females (*n* = 114), while having 26% less total fat mass when expressed as a percentage of body weight.^39^ Fracture rates also differ between males and females,^40,41^ which may be attributed to the higher bone mineral density (BMD) and content (BMC) in males compared to females,^40^ and have implications for performance and safety in weight-bearing and endurance sports. One study of height and weight-matched groups of 18-year-old males (*n* = 36) and females (*n* = 36) found significantly greater BMC for males at the femoral neck (11%), femoral trochanter (27%), femoral shaft (10%), total proximal femur (16%) and proximal tibia (13%).^42^ This study also showed significantly greater BMD in femoral neck (6%), femoral trochanter (6%), femoral shaft (7%) and proximal tibia (7%) in males compared to weight and height-matched females.^42^

## Effects of GAHT on serum sex hormone concentrations

Gender-affirming hormone therapies have clear effects on circulating sex hormone concentrations, with testosterone the focal hormone in sports, given its clear association with muscle strength and performance in cisgender males.^43^ Testosterone levels in healthy cisgender males (8.8–30.9 nmol/L) are four to fivefold greater than the upper end of the typical cisgender female range (0.4–2.0 nmol/L) and contribute significantly to biological sex differences.^44^ In males, testosterone influences athletic performance by promoting the development and maintenance of muscle mass and reductions in body fat.^45^ In females, this relationship is less clear, with mixed results in the literature.^46^ While evidence suggests testosterone is not a perfect proxy for performance, many sporting eligibility guidelines continue to focus on testosterone concentrations in both men and women. Despite limitations in using testosterone thresholds in sports, there is good evidence supporting the efficacy of feminizing therapies in lowering testosterone concentrations to the typical female reference range in trans women, and masculinizing hormone therapies in increasing serum testosterone concentrations to the typical male reference range in trans men.^47^

Feminizing gender-affirming hormone therapies involve oestrogen administration, often in combination with anti-androgen agents such as cyproterone acetate (CPA), spironolactone or gonadotropin-releasing hormone analogues (e.g. leuprolide).^47^ A 2020 systematic review found that anti-androgens CPA, medroxyprogesterone acetate (MPA) and leuprolide may be more effective than spironolactone at suppressing serum total testosterone concentrations when combined with oestrogen, but this is likely because of differences in the mechanism of action with spironolactone blocking peripheral androgen receptors rather than reducing testosterone production.^48^ For example, a 2019 cross-sectional study of trans women using GAHT for a median of 1.5 years found those treated with oestradiol plus CPA (median dose 50 mg daily) had significantly lower median serum total testosterone concentration (0.8 nmol/L), compared to those on oestradiol plus spironolactone (2.0 nmol/L: median spironolactone dose 100 mg/day) or oestradiol alone (10.5 nmol/L: median oestradiol dose 6 mg/day). In this study, the median testosterone concentration in both CPA and spironolactone groups was within the typical female range (0.4–2.0 nmol/L).^49^ Another study of trans women (*n* = 40) receiving transdermal oestradiol (1–2 mg/day) with either 50 mg/day of CPA (oral) or 3.75 mg/month of leuprolide (intramuscular injection) reported that both therapies reduced serum total testosterone concentrations to within the typical female range following 12 months.^50^ A 2021 study examined the effects of different doses of CPA paired with oestrogen in trans women (*n* = 882, finding not only that 10 mg of CPA was as effective as higher doses (25, 50, 100 mg), but that circulating testosterone concentrations reduced to within female reference range within 3 months.^51^ Comparatively, a 2019 study found circulating testosterone concentrations of trans women fell within the female reference range after 4 months of oestradiol treatment (*n* = 11).^52^ In a sports context, these findings suggest that trans women may therefore meet set testosterone thresholds at different times in their treatment, depending on what treatment is being followed. However, it is important to highlight the limitations of sporting guidelines which enforce testosterone limits on trans women. Given that anti-androgens are commonly used in feminizing GAHT, it cannot be assumed that testosterone concentrations directly reflect testosterone activity in trans women. Anti-androgens work to both suppress testosterone production and inhibit testosterone activity at the receptor level. A trans woman using anti-androgens may therefore present with high testosterone concentrations during the early stages of GAHT, but the action of testosterone is being inhibited at the critical receptor level.

In comparison to feminizing GAHT, masculinizing GAHT involves testosterone administration, either intramuscular, transdermal, or subcutaneous and leads to serum testosterone concentrations increasing to within the typical cisgender male range (8.8–30.9 nmol/L) following 12 months.^53–55^ A 2015 study in trans men (*n* = 23) found 12 months of masculinizing GAHT (1000 mg intramuscular testosterone undecanoate every 12 weeks) led to serum testosterone concentrations increasing from 0.9 to 21.8 nmol/L.^53^ A later 2018 study (*n* = 50 trans men)^54^ reported that intramuscular injections of testosterone undecanoate (1000 mg at weeks 0 and 6, then every 12–16 weeks) or testosterone enanthate (250 mg every 3–4 weeks) also increased serum testosterone concentrations to within male ranges within 12 months, from 1.4 to 18.3 nmol/L and 1.9 to 18.0 nmol/L in the testosterone undecanoate and testosterone enanthate groups, respectively. A further randomized controlled trial found that testosterone gel (50 mg/day) was just as effective as injections of testosterone undecanoate (1000 mg at weeks 0 and 6, then every 12–16 weeks) and testosterone enanthate (100 mg every 10 days) at increasing total testosterone concentrations to within normal male range by week 30 of treatment.^55^ While there are no current restrictions placed on men around testosterone concentrations, sport guidelines (e.g. FINA^20^ and World Rugby^56^) typically require trans men using testosterone (i.e. considered to be performance-enhancing substances) to obtain a Therapeutic Use Exemption (TUE) to allow them to use to testosterone whilst competing. Without a TUE, trans men competitors risk committing an Anti-Doping Rule Violation and being suspended from their sport.

## Effects of GAHT on body composition

**Figure 1.**
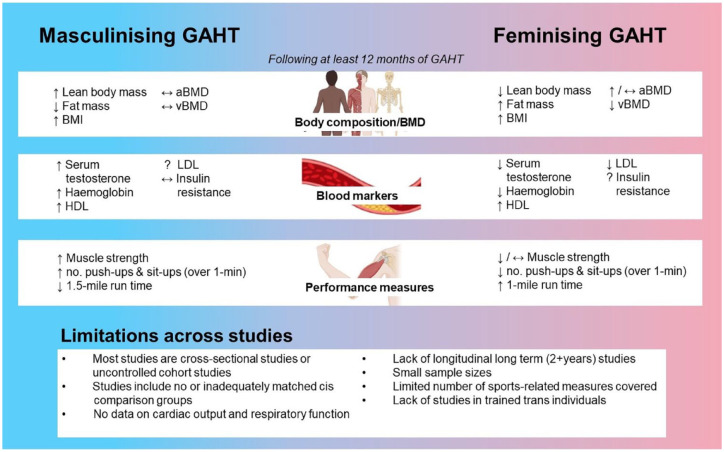
Summary and limitations of reported effects of GAHT on outcomes relevant to sports. aBMD, areal bone mineral density; BMI, body mass index; DMPs, differentially methylated positions; GAHT, gender-affirming hormone therapy; HDL, high-density lipoprotein; LDL, low-density lipoprotein; vBMD, volumetric bone mineral density.

Current evidence indicates that feminizing GAHT leads to increased fat mass and decreased lean mass in trans women (Figure 1), with inverse effects shown in trans men using masculinizing GAHT.^57–63^

A recent systematic review found that feminizing hormone therapies decreased lean body mass by 0.8–5.4% following 12 months.^57^ However, it is worth noting that is large variations in body composition changes are seen across individual studies. One study finds no change in body composition of 9.4% of trans women (*n* = 288) following 24 months of GAHT.^64^ A further prospective study in trans adolescent girls (*n* = 21) found a reduction of 4.7% in lean mass over 10.6 months (*n*_range_ = 5–31 months).^65^ These changes differ by region, with a prospective study (*n* = 179 trans women) finding the largest lean mass decreases occurred in the arm (−5 to −7%) and leg region (−3 to −4%), compared to gynoid regions (−2 to −3%) and android regions (−1% to +2%).^58^ In terms of total body fat, increases of 9–32%^58,66–68^ have been observed following 12 months of feminizing GAHT, with the greatest increases seen in the leg region (+37 to +47%), and the smallest changes in the trunk region (+16 to +25%).^58,65^ A study in trans women (*n* = 11) found increased regional body fat of 18, 27.4 and 27.2% in the trunk, legs and gynoid region, respectively, and an average 7.2% increase in total body fat of 7.2% following 6 months of feminizing GAHT.^69^ In terms of muscle area, a retrospective cohort study of trans women (*n* = 17) reported average reductions of 10% and 12% following 1 and 3 years of feminizing GAHT, respectively.^70^ Further research reports an increase in body mass index (BMI) in trans women following GAHT, with three studies reporting increases of 9–10% over 1–7 years of GAHT use.^59–61^ A limitation of prior studies exploring body composition changes in trans women is that they lack adequate comparison groups of cis women, making it difficult to assess how body composition may differ across cis and trans women. Existing reference standards for body composition measures indicate that males and females differ by an average of +10-12% across all age groups in mean % lean body mass^71^ and −20% for fat mass.^72^ Acknowledging this, prior research indicates that trans women using GAHT, on average, increase their total fat mass to within the expected female ranges within 12 months. In addition, trans women also experience decreases in lean body mass, which, on average, remain higher than expected female values after 12 months. However, these comparisons do not account for differences in age, height and BMI, which need to be considered for accurate comparisons. These measures also determine body composition outcomes in trans women, with, for example, higher BMI at start of hormone therapy related to smaller increases in body fat and larger decreases in lean mass.^58^

In comparison to feminizing GAHT, masculinizing GAHT leads to decreased fat mass and increased lean mass in trans men, with variation in degrees of change seen across individual studies. A prospective study examining the effects of GAHT over 12 months (*n* = 23 trans men) found a 10.4% mean increase in lean mass and a 9.7% mean reduction in total body fat mass,^53^ with comparable findings seen in a 12 months prospective study (*n* = 53 trans men, −13% fat mass, +11% lean mass),^66^ but smaller changes seen in a retrospective study (*n* = 121) (+3% lean mass, −3% fat mass) following 1.0 years of gonadotropin-releasing hormone analogues (GnRH) and 2.4 years GAHT.^68^ In trans adolescents, one prospective study found an 8.6% increase in lean mass following a mean 11.6 months (*n*_range_ = 4–40 months) of treatment in trans boys (*n* = 44).^65^ Another prospective study (*n* = 162 trans men) reported similar findings, with a mean 10% decrease in total body fat and a 10% increase in lean mass over 12 months of GAHT. This study observed the largest body fat decreases in the leg (−14% to −19%) and gynoid regions (−12% to −16%), and the largest lean mass increases in the arm region (+18 to +21%).^58^ Prior research also found that the BMI of trans men significantly increases following 6 months,^60,62^ and 12 months of GAHT^55^ with increases of 9–10%, and 2% reported at these time points, respectively. Limited studies have gathered comparison data from cis men and women. A 2012 study in trans men (*n* = 50) and cis women (*n* = 50) matched for age, height and BMI found that, on average, trans men had 30% less body fat and 9% more lean body mass compared to cis women. A limitation of this study was that it assessed trans men who had undergone gender affirmation surgery in addition to long-term GAHT and therefore, the separate effects of GAHT and gender confirmation surgery on body composition measures could not be determined. A more recent 2022 study compared body composition, among other measures, between trans men (*n* = 19) and cis men (*n* = 19) matched for age and BMI, and found trans men had 26.3% lower total lean mass and 18% higher body fat than cis men (*n* = 19), following a minimum of 6 months of GAHT.^63^ A retrospective study examined muscle area in those undergoing masculinizing GAHT (*n* = 19), finding increases of 16% and 15% following 1 and 3 years of GAHT, respectively.^70^ This study however did not have any comparison groups, so data could not be compared to cisgender individuals. These findings indicate that trans men likely, on average, possess lower muscle mass and higher body fat than cis men. This has potential safety implications, such as increased risk of concussion or injury, for trans men in high-contact sports. These risks are already acknowledged in many sporting guidelines. For example, World Rugby requires trans men to provide a confirmation of physical ability to play, which includes written acknowledgement and acceptance by the player of the associated risks of playing contact sports with males who are statistically likely to be stronger, faster and heavier than them, as well as written confirmation from a medical practitioner or qualified coach that the player is in a physical condition to play.^56^

## Effects of GAHT on muscle strength

A 2020 systematic review reported decreased or no change in muscle strength following at least 12 months of GAHT, across five longitudinal studies (*n*_range_ = 11–249 trans women). The largest of these studies reported that grip strength decreased by a mean of −1.8 kg in trans women following 12 months of GAHT^73^; however, this study did not include a comparison to a cisgender cohort. A smaller longitudinal study (*n* = 20) of the same time frame found no change in grip strength following 12 months GAHT in trans women,^67^ and a more recent 2021 prospective study (*n* = 11) found a reduction of 7.7% in grip strength over a shorter 6 month time frame.^69^ Conversely, a 2020 longitudinal study reported that muscle strength, determined using isokinetic dynamometry, was maintained in trans women (*n* = 11) following 12 months, with muscle strength greater in trans women, compared to height-adjusted cis women (*n* = 14).^52^ Limitations of this study were in its small sample size, with differences in age and body size between groups not adjusted for. Furthermore, the study and the 2020 systematic review were limited to 12 months, with potential further reductions in strength after this time was not captured. Cross-sectional analyses of trans women (*n* = 23) using GAHT for longer than 12 months (mean duration of 8 years), found that trans women had lower peak quadriceps strength (−25%), peak bicep strength (−33%) and grip strength (−23%) compared to height-matched cis men.^74^ However, this study did not include comparisons with matched cis women and did not match participants for physical activity level or training status. In addition to the limitations mentioned above, another limitation of prior studies is that the majority of studies are conducted in untrained trans women. A further cross-sectional study examining muscle strength using a hand grip dynamometer in trans women who had completed an average of 14.1 ± 3.5 years GAHT found that, on average, the muscle strength data of trans women fell below that of cisgender men but was significantly greater (16%) than the values of cisgender women.^75^ Most studies assessing muscle strength have utilized grip strength which has not been adjusted for hand size and overall is unlikely to be a good reflection of overall physical performance. Further, only two related studies have examined athletic performance in athletic trans people. A 2021 retrospective analysis of fitness tests performed by the United States military commencing GAHT during service was the first study to examine GAHT-related changes in trained trans individuals.^76^ Before beginning GAHT, military trans women (*n* = 46) performed 31% more push-ups and 15% more sit-ups in 1 min compared to cis women (*n* > 500,000), with these differences disappearing after 2 years of GAHT.^76^ A later study that drew from a larger sample set of US military personnel (*n* = 228 trans women) with extended follow up, found similar trends for the sit-up and push-up performance of trans women to decline steadily alongside months using GAHT.^77^ However, compared to cisgender women, trans women performed significantly better in sit-ups and push-ups, up until 4 years of GAHT, where no statistically significant difference was found in sit-up performance between trans and cisgender women.^77^ Differences in findings across studies likely stem from differences in sample size and dropout and should be interpreted with caution given the large number of dropouts across both studies. Collectively, research is limited with few longitudinal studies beyond 2 years and a lack of prospectively recruited comparison groups. Results suggest a reduction in muscle strength after starting GAHT, but the duration to see significant differences is unclear.

In trans men, limited studies have examined the effect of GAHT on muscle strength measures. A prospective study reported that knee extension and flexion torque, which is the maximum force applied by a muscle that causes rotation, increased by 16% and 26%, respectively, in trans men (*n* = 12) following 12 months GAHT,^52^ with further studies finding significant increases (~18%) in the grip strength of trans men over 12 months.^53,73^ A more recent 2022 cross-sectional study compared the hand grip strength of trans men undergoing GAHT for 10–28 months (*n* = 19), and cis men (*n* = 19) and demonstrated that cis men had 40% greater hand grip strength than that of trans men.^35^ Further, a retrospective analysis of fitness tests performed by the United States military found that trans men (*n* = 29) performed fewer push-ups (43%) before GAHT commencement, compared to cis men (*n* < 2.3 million), with no differences between groups found following 1 year of GAHT. This study also reported that trans and cis men were comparable in sit-up performance prior to GAHT commencement, with the sit-up performance of trans men than exceeding that of cisgender males after 12 months of GAHT.^76^ A later study in an extended sample set of US military (*n* = 146 trans men^77^), demonstrated that trans men had lower performance in the push-up tests (−10%) compared to cisgender men, up to 2 years of GAHT, with greater performance in these tests, compared to cisgender, shown following 3 (+5%) and 4 (+15%) years GAHT.^77^ This study also reported better performance in the sit-up test at 1 year GAHT (+4%), relative to cisgender men, which rose to as high as 13% following 4 years GAHT.^77^ However, limitations of these studies include high variability in the time between baseline and follow-up periods in trans participants, fitness tests being performed in a non-controlled setting, and factors such as training volume, intensity or type were not accounted for.^76^ Further research addressing limitations is required to improve understanding of strength differences between trans and cis men and provide updated guidance for participation in high-impact sports that rely on muscle mass and strength.

Alterations in muscle at a cellular or tissue level may partially underpin observed physiological changes or differences in muscle strength between cisgender individuals and trans individuals using GAHT. In this regard, the theory of ‘muscle memory’^78^ has been increasingly discussed in relation to trans women athletes. Muscle memory theorizes that muscle retains the capacity to perform tasks it has previously undergone, with suggestions that trans women, therefore, may retain muscle strength advantages over cis women after transition due to cellular or epigenetic marks retained from prior life exposures to testosterone and myonuclei retention. However, it is important to highlight that this area has not been explored in those undergoing GAHT. Myonuclei retention plays a potential role in muscle memory,^79^ with prior research showing myonuclei numbers are associated with training and testosterone use.^79,80^ In mice, a 2010 study in mice^79^ found muscle fibre myonuclei increased after exercise, with the acquired myonuclei retained during the detraining phase. Similar findings were found in female mice treated with testosterone propionate, with a 66% increase in myonuclei content observed following 14 days and a 10% elevation in myonuclei remaining across the mouse lifespan.^81^ In humans, a 1999 study compared the muscle physiology of powerlifters who were users (*n* = 9) and non-users of high-dose steroids (*n* = 10) and found that the number of myonuclei and the proportion of myonuclei were significantly higher in the steroid users.^80^ However, the concept of muscle memory has mainly been based on animal models, with a recent comprehensive review^82^ and systematic review and meta-analysis^83^ finding no clear evidence for the existence of muscle memory by myonuclei retention in both animal and human studies. In particular, it appears that ‘muscle memory’ is a phenomenon that is not observed in many studies or not retained long term, with many studies,^82,83^ and it could be debated that any memory marks altered during previous training would be easily modified or overridden by changes in sex hormone concentrations and the subsequent systematic effects of GAHT. Further research exploring physiological and epigenetic changes in trans men and women using GAHT could provide great molecular insights into the performance differences between cis and trans populations and is an area of future need.

## Effects of GAHT on endurance performance measures

Little research to date has explored the effects of GAHT on endurance performance. Thus far, the evidence indicates that GAHT can influence markers of oxygen transport, such as haemoglobin.^57^ The oxygen-carrying capacity of haemoglobin plays a vital role in determining endurance performance, with maximal oxygen uptake (VO_2_max), the most robust measure of endurance performance, increasing by approximately 1% for each 3 g/L increase in haemoglobin.^84^ To date, one study has been performed assessing the effects of feminizing GAHT on VO_2_peak, with a cross-sectional study finding that trans women (*n* = 15) who had received GAHT for 14.1 ± 3.5 years, on average, had a VO_2_peak/weight (relative to bodyweight) that fell lower the mean VO_2_peak/weight for both cisgender men and cisgender women. However, significance was only found between trans women and cisgender men, not trans women and cisgender women.^75^ This study also found that for absolute VO_2_peak, trans women VO_2_peak was significantly higher than that of cisgender women (20%), and significantly lower than that of cisgender men (29%).^75^ A limitation of this study is in its cross-sectional design, with the VO_2_peak of individuals before GAHT commencement not known, and the degree of change in VO_2_peak following GAHT commencement not able to be investigated. A 2021 systematic review that evaluated nine haemoglobin-related longitudinal studies (*n*_range_ = 12–239 trans women) and six cross-sectional studies (*n*_range_ = 23–182 trans women) reported consistent reductions in circulating haemoglobin concentrations (−3.4 to −14%) following 3–36 months of GAHT in trans women.^57^ Findings of this systematic review further demonstrated that haemoglobin concentrations of trans women decreased to typical female ranges after 4 months GAHT.^57^ In trans men using GAHT for at least 12 months (*n* = 79), a cross-sectional study found that haemoglobin concentrations (12.8–17.4 mg/dL) were comparable to cis male reference ranges (13.0–18.0 mg/dL).^85^

Findings of GAHT-related changes in haemoglobin suggest gender-affirming hormones influence oxygen transport and uptake in trans individuals, with likely broader impacts on endurance performance. However, no studies have yet reported changes in robust and direct markers of endurance fitness (e.g. VO_2_max). A retrospective study of military personnel, previously described in the above muscle strength section, compared 1.5 mi run times between trans men (*n* = 49), trans women (*n* = 46) and cis military personnel not undergoing hormone therapy.^76^ This study reported that trans women had 21% faster running times than cis women before hormone commencement, with this difference reducing to 9% after 2 years of GAHT.^76^ No differences in running times were observed between trans men and cis men.^76^ A more recent study, which analysed a larger data set of this US military sample (*n* = 228 trans women, 146 trans men), with extended follow-up, found trends for 1.5 mi run times to decrease with GAHT in trans women, with run times statistically greater than cisgender women up until 2 years of GAHT. This study also found that, compared to cisgender men, trans men had statistically lower run times up until 3 years into GAHT.^77^ Differences in findings across these US military studies likely arises from differences in sample size and dropout, with a limitation of these studies being the large decrease in participants over the respective timelines, with the more recent study by Chiccarelli *et al.*^77^ seeing a 96% drop-off from baseline. However, as touched on in previous sections, these studies also have limitations in being retrospective analyses of fitness tests performed in a non-controlled environment, with confounders not considered in analyses. Overall, current data indicates haemoglobin concentrations are comparable between trans women and cis men, as well as trans men and cis men following several months of GAHT. It is unclear whether changes in haemoglobin reflect changes in endurance performance, with a clear lack of studies exploring this area. A focus of further research should be on evaluating potential changes in VO_2_max in trans individuals using GAHT, given that VO_2_max is a major predictor of performance in sports such as distance cycling and distance running,^37,86,87^ with clear sex differences in this measure reported.^36^ There is a clear lack of control for confounders in this area, with factors such as body size, muscle mass and fat mass not considered during analysis in previous studies.

## Effects of GAHT on bone health

Markers of bone health, such as BMD, may be influenced by GAHT, with implications for participation in weight-bearing sports. Bone has key functions in structural support and allowing efficient movement,^88^ with mechanical loading involved in physical activity has a role in bone development.^89^ Athletes who compete in weight-bearing sports, such as gymnastics, are found to have 5–15% higher BMD than non-athletes.^90^ Furthermore, high-impact activities, such as endurance running, are associated with a greater risk of bone stress fractures.^91^ This highlights the importance of bone health and strength for performance and safety in a range of sports.

A systematic review and meta-analysis conducted in 2019 (*n* = 812 trans women, *n* = 487 trans men), found no effect of GAHT on BMD at all sites evaluated (femoral neck, total femur and lumbar spine), except for the lumbar spine BMD for trans women, where a small but significant increase was observed after 12 and ⩾24 months of GAHT.^92^ However, this review concluded that current evidence is of low to moderate quality, with inconsistencies commonly reported across studies comparing BMD changes in trans and cis cohorts. For example, A 6-month prospective study in trans women (*n* = 11) reported lumbar spine BMD significantly increased by 3.9%, but changes in femoral neck and total femur BMD were not significant, and comparisons to cisgender cohorts were not made.^69^ A more recent 2022 cross-sectional study reported total BMD and femoral neck BMD was lower in trans men using GAHT for at least 6 months (*n* = 19), when compared to age and BMI-matched cis men (*n* = 19).^63^ In contrast, another cross-sectional study in trans men (*n* = 15) and women (*n* = 24) using GAHT for 2–25 years found BMD Z scores for trans men did not differ from age-matched cis male reference groups, except for distal tibial diaphysis BMD, which was lower in trans men.^93^ This study further reported whole body and distal tibial diaphysis BMD *Z* scores were higher in trans women compared to cis female reference values.^93^ Unclear findings likely reflect small sample sizes and studies having differing hormone regimes, durations and comparator groups. Furthermore, these studies were limited in assessing areal BMD using dual-energy X-ray absorptiometry, which does not provide information on volumetric and structural changes. Addressing this limitation, a cross-sectional study published in 2022 examined differences in bone microarchitecture and volumetric BMD (via high-resolution peripheral quantitative computed tomography images) between trans and cis cohorts matched by age (trans men: *n* = 41, trans women: *n* = 51, cis women: *n* = 71, cis males: *n* = 51). This study found trans men had significantly higher total volumetric BMD (+0.63 SD) and greater trabecular thickness (+0.38 SD) than cis women. Relative to cis males, trans women had lower total (−0.68 SD) and cortical volumetric BMD (−0.70 SD), and higher cortical porosity (+0.70 SD). Findings suggest trans women experience deteriorated bone microarchitecture with GAHT, with bone microarchitecture not compromised in trans men.^94^ However, additional prospective longitudinal studies are needed to confirm findings. Given BMD correlates with measures of muscle strength in weight-bearing sports and fracture risk in endurance sports,^90,91^ this is an area requiring further investigation and consideration in sports guidelines.

## Effects of GAHT on metabolic and disease risk markers

Gender-affirming hormones may influence risk factors for cardiometabolic diseases, such as blood lipids and insulin resistance.^28,95^ This is of relevance to sports participation at both elite and community levels, given regular participation in sports and exercise plays an important role in improving and mediating cardiometabolic health.^96^

An analysis of United States survey data (*n* = ~2.7 million adults, including ~3000 trans adults) reports a two to fourfold increase in the rate of myocardial infarctions in both trans men and women, compared to cis population,^97^ with a further cross-sectional analysis of ~4000 trans adults and cis reference cohorts (n ~100,000) finding a significantly higher incidence of venous thromboembolism in trans women.^98^ A 2019 systematic review^28^ found varied evidence of GAHT-related changes in cardiometabolic risk markers, including high-density lipoprotein (HDL) concentrations and low-density lipoprotein (LDL) concentrations. In studies of trans women, increases in HDL concentrations were reported (14-27% over 6–24 months GAHT), with decreases in LDL concentrations (8-13% from 6 to 18 months GAHT).^99–102^ In trans men, HDL concentrations increased in several studies (3-33% over 6–24 months of GAHT), but LDL findings were inconclusive.^99–104^

A systematic review of 26 studies (*n* = 751 trans men, *n* = 689 trans women) reported that masculinizing GAHT has no impact on insulin resistance in trans men but that feminizing GAHT may exacerbate insulin resistance in trans women.^95^ However, a 2022 retrospective study compared the incidence of type 2 diabetes in adult trans women (*n* = 2585) and trans men (*n =* 1514) with the general population rates from their birth-assigned sex and found no significant difference in the incidence of diabetes.^105^ Therefore, while the current evidence suggests GAHT may impact insulin resistance, this does not seem to confer a greater risk of type 2 diabetes. Additional long-term controlled studies are required, with current evidence largely based on uncontrolled studies of short duration (<2 years) and findings likely confounded by lifestyle factors such as physical inactivity ^23^ and alcohol and nicotine use^106^ in trans individuals.

## Limitations and future directions

Current knowledge on the sports implications of gender-affirming hormone therapies is lacking (Figure 1). This is an emerging field of growing interest in health and sporting fields, with clear limitations in the existing literature. There is a clear lack of long-term longitudinal studies (2 + years), and therefore limited data and information on the long-term performance changes in both trans men and trans women using GAHT. Further, many studies to date have been cross-sectional or uncontrolled cohort studies that do not include accurate comparator groups of cis participants matched for important attributes such as age, height and BMI. Matching for these factors in research studies is needed to more robustly evaluate the effect of GAHT on the measures of interest, separate to possible confounding factors, and to subsequently avoid over and underestimating the influence of GAHT. GAHT-related changes in testosterone concentrations and body composition are the most researched areas, with these measures not perfect indicators of athletic capacity. Current evidence covers a limited range of performance measures (i.e., muscle strength), with future directions in investigating important markers of fitness (e.g. VO_2_max, cardiac output, maximal power output, W_max_/body weight ratio) and muscle function (e.g. epigenetics, muscle enzyme activity, fibre type). Of relevance is a well-controlled prospective matched cohort study (the GAME Study) just commenced by the authors that aim to address this limitation and examine a wider range of performance and muscle measures.^107^ Further, it is notable for highlighting that no studies exist comparing fitness and performance measures between trans individuals who commenced before and after the onset of puberty, and the related effects of puberty blockers; this is an area of future research, given recent policies made by FINA to exclude trans people from competing in elite competitions, unless they began GAHT pre-puberty,^20^ it is of interest to understand the effects of puberty blockers on measures of sports performance. Importantly, most studies have been conducted in untrained trans cohorts, with it was unclear whether findings are generalizable to athletes. The 2021 IOC framework on Fairness, Inclusion and Non-Discrimination on the Basis of Gender Identity and Sex Variations^8^ states that sporting guidelines should be based on robust and peer-reviewed research and largely based on data gathered for groups with appropriate athletic engagement and measures appropriate to specific sport disciplines. This is not the current level of evidence available, and limitations in current knowledge should therefore be highlighted in current sporting guidelines and policy actions to prevent stigma, discrimination and potential harm to the trans community. Sporting organizations and federations should be cautious in implementing exclusive policies, particularly with a lack of trans people at the elite level, and in the absence of evidence, an individualized approach to the assessment of trans people should be taken.

## Conclusion

The present literature indicates feminizing GAHT effectively lowers testosterone concentrations to the typical female ranges in trans women after 12 months, with masculinizing hormone therapies increasing concentrations to the male range in trans men. Feminizing GAHT is further shown to increase fat mass and decrease lean mass in trans women, with opposite effects observed in trans men using masculinizing GAHT. In trans women, muscle strength measured in small studies is shown to decrease or not change following at least 12 months of GAHT and appears to continue to decrease beyond 12 months, with increased strength observed in trans men using GAHT. Longer durations of GAHT appear to be associated with significantly lower muscle strength compared to cis men. Studies comparing strength measures between trans and cis women report unclear results, which are dependent on study design, sample size and the strength outcomes measured. Little research has explored the effects of GAHT on endurance performance, but from the current literature, endurance performance is impaired in trans women (based on 1.5 mi run and VO_2_max data), and is somewhat improved in trans men (based on 1.5 mi run time data). Further, consistent changes in markers of oxygen transport (haemoglobin) suggest GAHT influences oxygen capacity; however, further investigations are required. Prior research reports varied GAHT-related changes in HDL, LDL and insulin resistance, with data limited due to a lack of long-term longitudinal studies and likely confounded by lifestyle factors. Overall, current limitations of this field include a lack of long-term studies, small sample sizes and the use of no or inadequate comparison groups. Future directions should investigate a broader range of markers related to fitness, and muscle health and functioning. There is a need for well-controlled longitudinal studies on GAHT in non-athletic and athletic trans people to address the current limitations and provide more robust data to inform inclusive and fair sporting programmes, policies, and guidelines.

## Declarations

**Ethics approval and consent to participate:** Not applicable.

**Consent for publication:** Not applicable.

**Availability of data and materials:** Not applicable.
